# Fellow eye comparison between alcohol-assisted and single-step transepithelial photorefractive keratectomy: late mid-term outcomes

**Published:** 2020

**Authors:** Alexander Harold Rodriguez, Virgilio Galvis, Alejandro Tello, Margarita María Parra, Marcela Ángela Rojas, Mosquera Samuel Arba, Anthony Paul Camacho

**Affiliations:** * Ophthalmology Department, Universidad Industrial de Santander UIS, Bucaramanga, Colombia; ** Ophthalmology Department, Fundación Oftalmológica de Santander FOSCAL, Floridablanca, Colombia; *** Centro Oftalmológico Virgilio Galvis, Floridablanca, Colombia; **** Ophthalmology Department, Universidad Autónoma de Bucaramanga UNAB, Floridablanca, Colombia; ***** Biomedical Engineering Office, Research and Development, SCHWIND eye-tech-solutions GmbH, Kleinostheim, Germany

**Keywords:** refractive surgical procedures, photorefractive keratectomy, excimer laser, visual acuity, contrast sensitivity

## Abstract

**Objective:** To compare late mid-term results of two different surgical approaches of surface excimer laser ablation for myopic and astigmatic errors in contralateral eyes of the same patients.

**Methods:** Prospective cohort study. A photorefractive keratectomy technique was performed on the right eye and single-step transepithelial photorefractive keratectomy on the left eye of the same patient, in 2012. Postoperative uncorrected and corrected visual acuities, manifest refraction, contrast sensitivity, objective scatter index, tear film stability assessed by serial measurements of objective scatter index and aberrometry as well as occurrence of haze, were compared between groups of eyes.

**Results:** Thirty-two eyes of 16 patients with a mean time of follow-up of 35.2 +/ - 5.0 months (range 30-46 months) were evaluated. No significant differences were observed in postoperative results (visual acuity, spherical equivalent, defocus equivalent, higher-order aberrations, objective scatter index, tear film stability and contrast sensitivity). Contrast sensitivity tended to be better in transepithelial photorefractive keratectomy technique, under photopic lighting conditions without glare and mesopic conditions both with glare and without glare, however, no statistically significant differences were found. No eye presented corneal haze at the last examination.

**Conclusion:** No statistically significant differences in visual acuity, refractive results, contrast sensitivity, objective scatter index, tear film stability or ocular aberrometry were observed between the two surface ablation techniques.

## Introduction

Surface ablation techniques, such as Alcohol-assisted Photorefractive Keratectomy (PRK), Transepithelial Photorefractive Keratectomy (TransPRK) and Laser-Assisted Subepithelial Keratomileusis (LASEK), have shown to be effective and safe alternatives for refractive corrections and without significant differences neither between them nor when comparing them with the techniques that include the cutting of a corneal flap [**1**-**4**]. Laser epithelial debridement previous to ablation of the stroma has been used from early 1990, but the technique was not popularized because it was a two-step procedure and, in addition, either the laser de-epithelization was not complete and a supplementary mechanical removal of the remnant epithelium was needed or the surgeon had to use various subtle signs (such as the dissipation of the autofluorescence of the epithelium) to determine when epithelial ablation was complete [**5**-**10**]. It was not until 2009 that a system was developed to perform epithelial and stromal ablation in one step in a predictable manner (Schwind Amaris, Schwind eye-tech-solutions GmbH, Kleinostheim, Germany) and this approach has been reported to have similar refractive results as PRK and possibly some advantages like less post-operative pain, less epithelial erosion-related symptoms, shorter epithelial closure time and in some studies less corneal haze [**11**-**26**]. The purpose of the present study was to evaluate the outcomes, comparing alcohol-assisted PRK and single step TransPRK techniques, considering refractive results, aberrometry, contrast sensitivity test, Objective Scatter Index (OSI, measured by AcuTarget®, Visiometrics SL, Cerdanyola del Vallès, Spain) and tear film stability assessed by serial measurements of OSI with a late mid-term follow-up time (minimum 30 months and a mean of 35.2 +/ - 5.0 months) using a contralateral (fellow eye) approach.

## Materials and methods 

This prospective cohort study included patients with myopic refractive errors, who underwent ablation on the surface of the cornea with excimer laser using two different techniques: PRK technique in the right eye and single-step TransPRK in the left eye, in 2012, at a tertiary ophthalmological center in Bucaramanga, Colombia, by a single surgeon (VG). The study followed the tenets of the Declaration of Helsinki and was approved by the institutional ethics committee.

Patients with preoperative CDVA better than 20/ 30, older than 19 years of age, with refractive stability at least during one year and who had discontinuously worn soft or hard contact lenses for at least 2 and 4 weeks (respectively) prior to preoperative assessment, were included in the study.

Patients with history of autoimmune diseases, planned ablation depth greater than 100 μm and pachymetry thinner than 490 μm, were excluded. Patients with findings of corneal ectasia at corneal tomography, amblyopia or other ocular pathologies were not recruited either. 

**Surgical procedures:**

All surgical procedures were performed by a single surgeon (VG). In PRK technique, after proparacaine (5 mg/ ml) was instilled on the eye, a well filled with ethyl alcohol (200 mg/ mL) was placed on the central 9.5 mm of the cornea for exactly 30 seconds and afterwards the cornea was flushed with balanced saline. Then, an epithelial debridement was performed using an angled spatula. 

In single step TransPRK technique, laser ablative surgery was performed by removing sequentially in one step corneal epithelium and stroma using the algorithm included in the Schwind Amaris platform (ablating 55 microns centrally, and 65 microns peripherally, in addition to the refractive stromal ablation, which corresponded approximately to epithelial thickness). 

In both groups of eyes, laser photoablation was done with the Schwind Amaris 750S excimer laser (Schwind eye-tech-solutions GmbH, Kleinostheim, Germany) with a pre-established optimized algorithm that aimed to maintain the preoperative levels of higher order aberrations basically unaltered, avoiding induction of new aberrations (mainly spherical aberration), known as “Aberration-Free™” ablation algorithm. The optical zone was 6.5 mm in all eyes. 

Mitomycin C 0.2 mg/ mL was applied on the ablated stroma for 30 seconds in all the eyes, followed by irrigation with balanced saline solution, and then application of moxifloxacin (5 mg/ ml). A silicone hydrogel contact lens was finally placed on the cornea for 5 to 6 days. Postoperatively, patients received topical moxifloxacin, prednisolone and carboxymethylcellulose. 

**Outcome measures:**

The last evaluation was performed on patients between 30 and 46 months after the surgery, and the postoperative results assessed at the last check-up visit were analyzed. Outcome measures included uncorrected distance visual acuity (UDVA), corrected distance visual acuity (CDVA), manifest refraction, contrast sensitivity, Objective Scatter Index (OSI, measured by AcuTarget®) [**27**-**29**], tear film stability assessed by serial measurements of OSI [**30**], aberrometry, and safety and efficacy indices, which were compared between the study groups. Presence and grading of stromal haze were also analyzed.

Visual acuity (VA) was measured with an ETDRS chart, and then converted to LogMar notation for statistical analysis. Refractive results were analyzed using both spherical equivalent (SE) and defocus equivalent calculated from the manifest refraction [**31**,**32**].

The efficacy and safety indices were calculated as it follows: 

*- Safety index:* The ratio of postoperative CDVA, previously converted to decimal notation, to preoperative CDVA, previously converted to decimal notation, was determined for each eye. Then, the mean and standard deviation of those values were found for each group (PRK and single-step TransPRK).

*- Efficacy index:* The ratio of postoperative UDVA, previously converted to decimal notation, to preoperative CDVA, previously converted to decimal notation, was determined for each eye. Then, the mean and standard deviation of those values were found for each group (PRK and single-step TransPRK).

Postoperative contrast sensitivity was measured using a system with microprocessor-controlled glare and luminance level (Optec® 6500, Stereo optical Company Inc., Chicago, IL, USA) under mesopic (3 cd/ m2) and photopic lighting conditions (85 cd/ m2), with glare and without glare. 

Additionally, postoperative ocular aberrometry (KR-1W Wavefront Analyzer®, Topcon, Tokyo, Japan) was analyzed. 

The intraocular light scatter was quantified postoperatively through the optical quality analysis system AcuTarget® (Visiometrics SL, Cerdanyola del Vallès, Spain) to calculate the initial Objective Scatter Index (OSI) [**27**-**29**], then it was measured every 0,5 seconds for 20 seconds after in order to evaluate tear film quality [**30**]. 

**Statistical analysis:**

Statistical analysis was performed using Microsoft Excel® and Stata VE 12.0® with a significance level of 5%. Qualitative variables were summarized by absolute and relative frequencies. In contrast, quantitative variables were expressed by measures of central tendency (mean) and dispersion (standard deviation) according to the frequency distribution. Normality was considered by evaluating graphic behavior, asymmetry and kurtosis. Furthermore, a descriptive analysis was carried out to identify potential differences among preoperative findings between both groups using a Student’s t-test. Higher order aberrations, objective scatter index (OSI) and tear film stability values were compared using the Student’s t-test. Proportion of eyes achieving a given level of visual acuity were compared using the Fisher exact test. 

## Results

Sixteen subjects who underwent PRK in the right eye and single step TransPRK in the left eye, were evaluated. Mean age was 29.93 +/ - 7.58 years (range 20-53) and average follow-up time was 35.2 +/ - 4.9 months (range 30-46). 10 patients (62.5%) were men. 

Preoperative and postoperative visual acuity and refractive data are detailed in **Table 1**. Mean spherical equivalent (SE) in PRK-treated eyes was -2.11 ± 0.91 D, decreasing after the surgery to -0.23 ± 0.65 D. Similarly, TransPRK-treated group changed from a preoperative SE of -2.10 ± 0.71 D, to -0.05 ± 0.34D. Postoperative defocus equivalent values were 0.48 +/ - 0.64 D and 0.33 +/ - 0.4 D in the right and left eyes, respectively. No statistically significant differences were observed either in the postoperative SE (p= 0.39), defocus equivalent (p= 0.41) or in the surgically induced change on those values (p values of 0.65 and 0.90) at the last follow-up examination between the two groups of eyes.

**Table 1:** Preoperative and postoperative parameters in PRK and single-step TransPRK treated eyes

	Preoperative findings					Postoperative findings				Difference pre vs. postoperative			
Parameter	PRK (n=16 eyes)		TransPRK (n=16 eyes)			PRK (n=16 eyes)		TransPRK (n=16 eyes)			PRK (n=16 eyes)	TransPRK (n=16 eyes)	
	Mean ± SD	Range	Mean ± SD	Range	p value	Mean ± SD	Range	Mean ± SD	Range	p value	Mean+/- SD	Mean+/- SD	p value
UDVA LogMAR (Snellen)	0.79 ± 0.27 (20/147)	0.17 - 1.3	0.78 ± 0.24 (20/140)	0.39 - 1.3	0.913	0.05 ± 0.12 (20/23)	0.39 - 0 (20/20 - 20/50)	0.01 ± 0.03 (20/20)	0.09 - 0 (20/20 - 20/25)	0.206	0.74+/- 0.28	0.77+/- 0.23	0.743
CDVA LogMAR (Snellen)	0.006 ± 0.02 (20/20)	0 - 0.09 (20/20 - 20/25)	0.006 ± 0.02 (20/20)	0 - 0.09 (20/20 - 20/25)	1.000	0 (20/20)	0 (20/20)	0 (20/20)	0 (20/20)	1.000	0.006+/-0.02	0.006+/-0.02	1.000
Sphere (D)	-1.56 ± 1.03	-4.00 - 0	-1.66 ± 0.74	-2.75 - 0	0.755	-0.08 ± 0.63	-1.5 - 0.75	0.09 ± 0.37	-0.75 - 0.75	0.366	-1.48+/-0.91	-1.75+/-0.73	0.362
Cylinder (D)	-1.09 ± 1.13	-3.5 - 0	-0.80 ± 0.77	-3 - 0	0.403	-0.30 ± 0.41	-1.25 - 0	-0.28 ± 0.38	-1.25 - 0	1.000	-0.81+/-1.1	-0.52+/-0.62	0.366
Spherical equivalent (D)	-2.11 ± 0.91	-4.45 -0.75	-2.10 ± 0.71	-3.5 -1.0	0.973	-0.23 ± 0.65	-1.6 - 0.25	-0.05 ± 0.34	-0.88 - 0.25	0.390	-1.89+/-0.77	-2.01+/-0.71	0.65
Defocus Equivalent (D)*	2.66 ± 1.10	1 - 5.5	2.45 ± 0.87	1 - 4.25	0.554	0.48± 0.64	0 - 2	0.33 ± 0.40	0 - 1.25	0.410	2.17+/-0.97	2.13+/-0.84	0.902
* Defocus Equivalent = Absolute value of spherical equivalent + ½ absolute value of cylinder.							

**Fig. 1:**
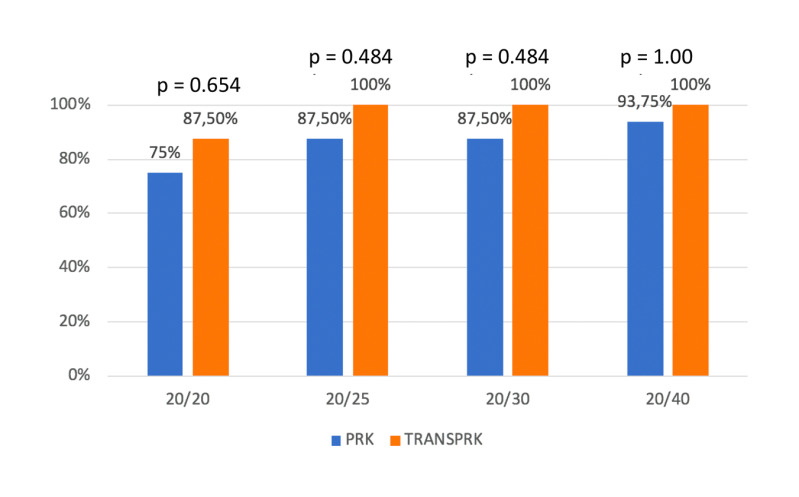
Late mid-term visual results in PRK and TransPRK treated eyes: Cumulative percentages of eyes attaining specified cumulative levels of UDVA at the last postoperative follow-up (mean= 35.2 months) in the 2 groups. No statistically significant differences were found (p values shown in the figure calculated using exact Fisher test)

75% of the eyes in the PRK group achieved postoperative UDVA of 20/ 20 at the last examination, and 87.5% of TransPRK-treated eyes reached UDVA of 20/ 20 postoperatively. No statistically significant differences were observed among the proportions of the eyes reaching specified cumulative levels of UDVA at the last postoperative follow-up (**Fig. 1**).

Efficacy index was 0.93 +/ - 0.19 and safety index 1.03 +/ - 0.09, for the PRK treated eyes, and 0.99 +/ - 0.05 and 1.02 +/ - 0.06, respectively, for TransPRK treated eyes. No statistically significant differences were observed between the two groups either in the efficacy (p=0.243) or in the safety indexes (p=0.569). 

The postoperative Objective Scatter Index (OSI) value at the last visit exhibited no significant differences between the two groups of eyes (p=0.734): in right eyes, treated with PRK, it was 0.62 +/ - 0.42 (range 0.1-1.6) and in left eyes, treated with TransPRK, 0.69 +/ - 0.72 (range 0.2-3.2). Tear Film stability (OSI measured during 20 seconds) yielded a mean of 1.44 +/ - 0.92 (range 0.45- 3.14) in the PRK-treated group of eyes and 1.19 +/ - 0.73 (range 0.48-2.65) in eyes treated with TransPRK, exhibiting no significant differences (p = 0.40). 

**Fig. 2:**
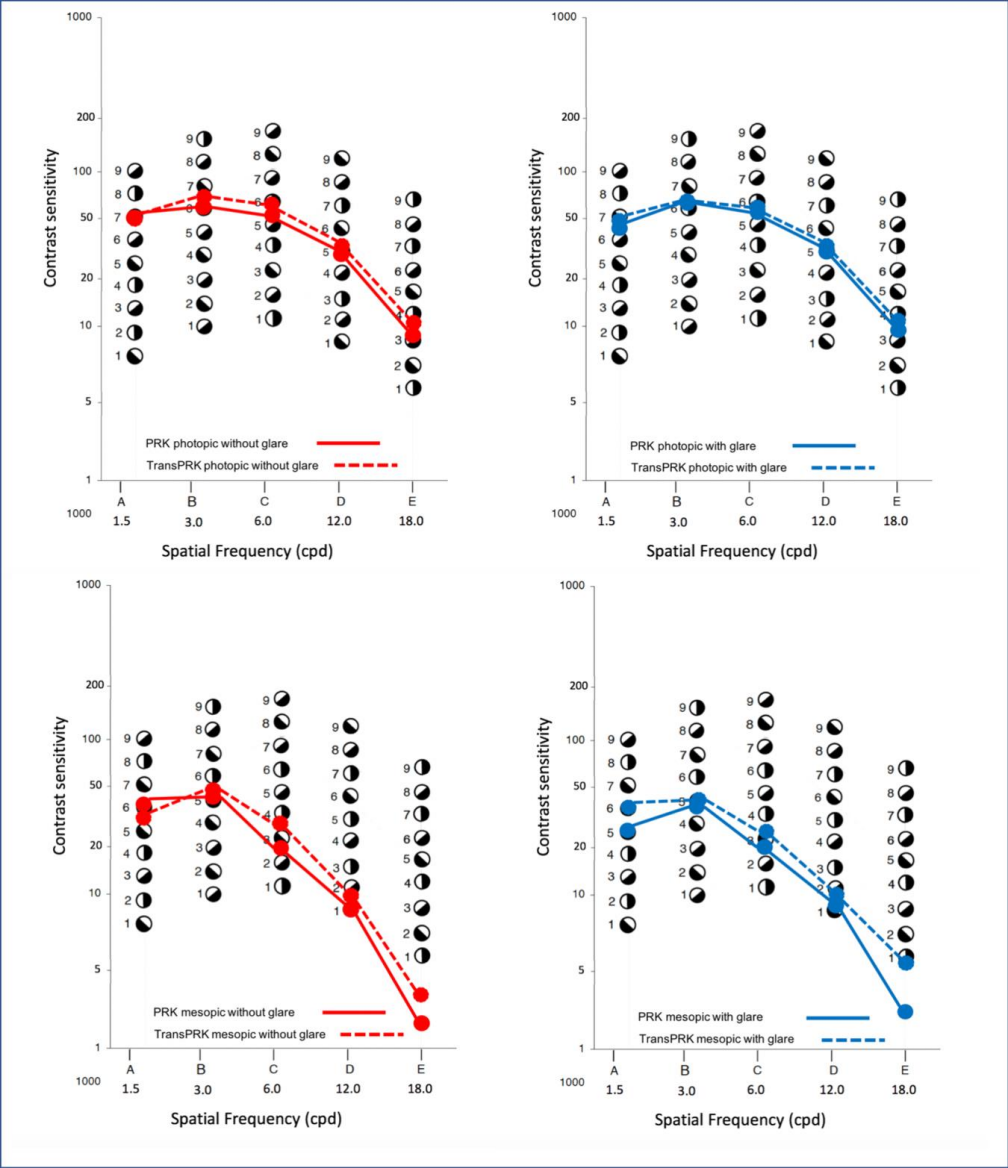
Contrast sensitivity at spatial frequencies of 1.5, 3.0, 6.0, 12.0 and 18.0 cycles per degree at photopic (superior row) and mesopic (inferior row) conditions, without (left) and with glare (right), at the last follow-up examination (mean:35.2 months after surgery). No statistically significant differences were found

No statistically significant differences were found with regard to postoperative contrast sensitivity between the two groups of eyes (**Fig. 2**). However, contrast sensitivity under photopic lighting conditions without glare, as well as in mesopic conditions with glare and without glare, revealed a slightly better performance in TransPRK-treated compared to PRK-treated eyes (**Fig. 2**), but as mentioned, the differences were not statistically significant (**Fig. 2**). 

Postoperative aberrometry measures at 35.2 months of follow-up (with natural pupil size) are shown in the **Table 2**. No statistically significant differences were observed between the two groups. However, root mean square (RMS) of coma, spherical aberration, and that of total higher order aberrations had values slightly lower in the TransPRK-treated eyes, but as mentioned, without reaching statistical significance. 

**Table 2:** Postoperative aberrometry measurements (Topcon’s KR-1W Wavefront Analyzer®)

Parameter	PRK (n=16 eyes)		TransPRK (n=16 eyes)		
	Mean	Range	Mean	Range	p value
Pupilar diameter	5.82 ± 0.65	4.55 to 7.20	5.99 ± 0.66	4.90 to 7.13	0.1332
Coma (μm)	0.27 ± 0.15	0.06 to 0.12	0.222 ± 0.171	0.04 to 0.56	0.1684
Trefoil (μm)	0.19 ± 0.15	0.06 to 0.56	0.223 ± 0.18	0.01 to 0.59	0.1717
Spherical aberration (μm)	0.07 ±0.12	-0.11 to 0.32	0.058 ± 0.137	-0.12 to 0.28	0.3227
RMS Higher order aberrations (μm)	0.45 ± 0.21	0.20 to 0.90	0.435 ± 0.229	0.19 to 0.87	0.8149
RMS Total aberrations (μm)	0.98 ± 0.47	0.44 to 2.10	0.934 ± 0.447	0.37 to 1.68	0.3936
*RMS: Root mean square*					

## Discussion

This study assessed postoperative visual outcomes after excimer laser surface ablation for correction of myopic refractive errors (both quantitative and qualitative), with an average follow-up time of 35.2 months (late mid-term), comparing PRK and single-step TransPRK techniques in contralateral eyes of the same patients. 

No statistically significant differences either in visual acuity or in refractive results (represented by sphere, cylinder, spherical equivalent and defocus equivalent) between PRK and TransPRK, were found, as already reported by other previously published studies [**3**,**4**,**11**-**13**,**17**,**19**,**23**,**25**]. However, other researchers have reported some better results using TransPRK (especially with the new refinements of the software) including better UDVA and CDVA results [**14**,**18**,**20**,**21**,**24**]. On the other hand, in 2015, Shapira et al. evaluated 3417 patients, showing that PRK-treated eyes demonstrated better refractive outcomes at 6 and 12 months after surgery (P < .0001). Nonetheless, the TransPRK was not a one-step surgery (as done using the Amaris Schwind system in the present study) but a two steps procedure with a different system, first using a PTK mode ablation at a depth of 50 μm followed by mechanical completion of deepithelization with a sponge [**15**].

The present study used the comparison approach between the two eyes of the same individual, and like, in the four published contralateral studies, which had a maximum postoperative follow-up of one year, there were no significant differences in refraction and visual results between PRK and one-step TransPRK treated eyes [**12**-**14**,**23**]. 

It is noteworthy that, although no differences were observed in the mean of UDVA between groups, according to cumulative visual acuity, in the present fellow eye study, there were lower percentages of eyes in the PRK group with UDVA better than 20/ 30 compared to TransPRK, although, as previously indicated, the differences were not statistically significant (**Fig. 1**). This finding was in contrast to the data reported by Luger et al., also in a contralateral eye study, who reported slightly more with visual acuities better than 20/ 20 at one year in the PRK-treated eyes [**12**]. 

Marginally better postoperative contrast sensitivity under photopic without glare, and mesopic conditions with glare and without glare in TransPRK-treated eyes were observed in the present study (**Fig. 2**) but differences were non-significant. Similarly, in their fellow-eye study, Luger et al. did not find any statistically significant difference in contrast sensitivity [**12**]. 

A slightly better performance in higher order aberrations at 35.2 months of follow-up in the TransPRK treated eyes was found in the present study, but it did not reach statistical significance. Similarly, no differences in aberrometry has been published [**13**,**22**]. 

In the present study, both contrast sensitivity and aberrometry were measured only at the last check-up visit after the surgery, so we could not evaluate the surgically induced change on those values, and could therefore induce a non-intentional bias if some of the eyes in one group had better or worse values of these parameters before surgery. This is a weakness of the present study. However, on the other hand, being a fellow-eye study, it is very probable that both eyes of the same patient had very similar characteristics on these parameters before the procedure. 

Intraocular scattering measurement, the objective scatter index (OSI), has been a recently implemented parameter for the determination of optical quality and has been used with that purpose after refractive surgery [**27**-**30**]. The OSI as determined by the AcuTarget® (Visiometrics SL, Cerdanyola del Vallès, Spain) using a laser (wavelength of 780 nm) with a double-pass technique (i.e. recording images from a single point source of light, after reflection in the retina and a double pass through transparent optic ocular media), represents the amount of scattered light. Some studies have shown its impairment initially after corneal procedures [**28**]. According to our knowledge, there has been no previous study comparing OSI in PRK and TransPRK. In our cases, we found no difference in the late mid-term postoperative OSI between groups of PRK-treated and TransPRK treated eyes. When determining the tear film stability by sequentially measuring OSI during 20 seconds of evaluation, results were slightly better in TransPRK-treated group, but the difference again was not statistically significant. 

With regard to corneal haze found at the late mid-term when the patients were examined the last time (i.e. between 30 and 46 months after surgery), none of the eyes showed clinically evident haze.

The small differences, not statistically significant, that we found in some postsurgical parameters when comparing eyes treated with PRK and those treated with single step Trans-PRK, were more probably not clinically significant either. Consequently, both techniques can be considered effective and safe for the correction of moderate myopic and astigmatic errors. Recently, Adib-Moghaddam et al. published a complete review on single-step TransPRK, and their conclusions are similar [**26**].

A limitation of the present study is the limited number of patients. Since the number of patients was small, the result was that although some of the observed differences could be real, they did not reach statistical significance. However, the importance of this work is based on the comparison of two different techniques in contralateral eyes, which allowed the homogenization features of studied samples, since many biological variables remained constant in both eyes, expecting to allow more reliable comparison in outcomes that represent the postoperative behavior of refractive surgery (v.gr. in contrast sensitivity performance), and in addition, to have the longest postoperative follow-up time that has been published in this type of comparative study (35.2 months) [**12**-**14**,**23**].

**Sources of funding**

This study did not receive any specific funding.

**Compliance with Ethical Standards**

The authors declare that they have no competing interest. All procedures performed in this study involving human participants were in accordance with the ethical standards of the institutional and/ or national research committee and with the 1964 Helsinki Declaration and its later amendments or comparable ethical standards. Informed consent was obtained from all individual participants included in the study.
